# Lateral Quadratus Lumborum Block versus Transversus Abdominis Plane Block in Laparoscopic Surgery: A Randomized Controlled Study

**DOI:** 10.1155/2022/9201795

**Published:** 2022-03-28

**Authors:** Omar Sayed Fargaly, Maged Labib Boules, Mohamed Ahmed Hamed, Mohammed Abdel Aleem Abbas, Mohammed Ahmed Shawky

**Affiliations:** Department of Anesthesiology, Faculty of Medicine, Fayoum University, Fayoum, Egypt

## Abstract

**Background:**

After laparoscopic abdominal surgery, we aim to evaluate the analgesic efficiency of US-directed bilateral transversus abdominis plane block (TAPB) and quadratus lumborum block (QLB).

**Methods:**

50 patients aged 18–60 years listed for elective laparoscopic abdomen operation were registered in this study. Cases were randomly allocated into two similar groups: TAPB and QLB groups. The first outcome was the growing morphine consumption on the 1^st^ day postoperatively. The second outcome involved VAS score, first analgesic necessities, and any postoperative complications. Statistical analysis was done with the 2-sample *t*-test, and Mann–Whitney U testing was utilized to compare medians for skewed end points. Qualitative data were introduced as numbers and percentages, and chi-squared testing was utilized to determine the significance.

**Results:**

The median cumulative morphine consumptions on the 1st day were high significantly in the TAPB group than in the QLB group (6 mg [6, 9] vs. 3 mg [3, 6], *p* value ≤0.0001]). The QLB group showed an increase in the median of the time to the first analgesic request in comparison with the TAPB group (17 hours [12, 24] vs. 8 hours [6, 24], *p* ≤ 0.001). In addition, on the 1st day, the mean VAS scoring at rest was lower in the QLB group.

**Conclusion:**

In comparison to the TAPB, the QL block delivers more successful pain relief, has an extended period of analgesic actions, extends interval to the 1^st^ analgesic necessity, is accompanied with lesser morphine consumptions, and may be utilized in multimodal analgesia and opioid-sparing regimens after that laparoscopic operation. This trial is registered with NCT04553991.

## 1. Introduction

Laparoscopic surgery is a common technique in many operations: cholecystectomy, appendectomy, inguinal hernia repair, hemicolectomy, sleeve, etc. [1]. Although postoperative pain after laparoscopy is lesser than in open operative techniques, the abdomen stiffness from the pneumoperitoneum (achieved as a step of the laparoscopic approach) and operative treatments may cause severe postoperative pain that will affect the patient satisfaction and the outcome of the surgery. [2] The pain was usually managed by opioids, leading to various side effects like vomiting, oversedation, nausea, and respiratory depression. [3] Pain is multifactorial and has significant interindividual variations, and the notion of adequate analgesia is a crucial opinion to recall. The inability to provide safe, adequate analgesia thereafter the abdomen operation is still one obstacle to introducing local anesthetic methods [4].

Numerous approaches are achieved to control the pain after laparoscopy. Lately, the TAPB defined by Rafi [5] was planned to compensate for the difficulties advanced by preexisting techniques. The TAPB is a previously recognized method and is a practical part of the multimodal pain management method for abdomen operations [6].

The quadratus lumborum block (QLB) is a recently defined local block that Blanco et al. primarily defined, which was concluded to give satisfactory analgesia for upper and lower abdomen operations [7]. There were many methods for QL block (posterior, lateral, transmuscular, and intramuscular) [8], but the block technique's difficulty is the main limitation. On the other hand, the intramuscular approach is straightforward, but its action mechanism is still unclear. This work aimed to compare and evaluate the analgesic effectiveness between QLB and TAPB after laparoscopic abdomen operations. The primary outcome was the collective morphine consumption on the 1st day postoperatively.

## 2. Patient and Methods

This prospective randomized, observer-blinded paralleled group research was performed after the tenets of the Declaration of Helsinki. This work was accepted by the local institutional ethics committee and the local IRB of Fayoum University Hospitals. This study is registered on ClinicalTrials.gov (NCT04553991; principal investigator: Mohammed Abdel Aleem; date of registration: 18/09/2020). Written informed agreement was attained from 50 adult patients listed for elective laparoscopy abdomen operations between July 2019 and February 2020. The current study adheres to the appropriate CONSORT strategies.

### 2.1. Inclusion Criteria

Patients listed for elective abdominal laparoscopic operations (inguinal hernia repairing, lost IUCD extracting, appendectomy, and ovarian vein ligation) were of ages between 18 and 60 yrs and were of American Society of Anesthesiologists Physical Status I or II.

### 2.2. Exclusion Criteria

The exclusion criteria were as follows: BMI >40, contraindications to local anesthesia (coagulopathy, severe thrombopenia, allergy to local anesthetic, and infections at puncture location), sepsis, chronic pain disorders that necessitate the intake of opioids at home, and any substantial neurological, cardiovascular, or breathing disorder.

### 2.3. Randomization and Blinding

Patients were arbitrarily divided into two groups (QLB group *n* = 25 and TAPB group *n* = 25) via computer-produced arbitrary numbers kept in distinct opaque packets unlocked by the authors just earlier the block. Thus, the patients and the statistics collectors were uninformed of the group distribution till the work's termination.

### 2.4. Preoperative Preparation

Regarding the local protocol considered to assess the patients, preoperative evaluation (history, examinations, and analysis) was performed. Preoperation, the contributors were taught about the VAS score (0–10) (0 = no pains and 10 = worst comprehensible pains) and the specifics of nerve block operations.

### 2.5. Anesthetic Management

On coming to the operation area, usual monitors (pulse oximeter, capnography, noninvasive BP monitor, and electrocardiogram) were utilized and sustained during the surgery. An 18-gauge marginal intravenous (IV) cannula was implanted, IV midazolam 0.03 mg kg^−1^, metoclopramide 10 mg, and ceftriaxone 1 gm have been managed to all cases as pre-medication, and then preoxygenation with O_2_ 100 percent for at least 3 minutes inductions of anesthesia was done with fentanyl 1*μ*gkg^−1^, propofol 1.5 to 2 mg kg^−1^, and atracurium 0.5-mg kg^−1^. Anesthesia was preserved via volume-controlled ventilation (VCV) tidal size 6–8 ml kg^−1^ with oxygen: air (50 : 50) with EtCO_2_ ≈ 35–40 mm Hg, isoflurane 1 : 1.5 percent volume concentrations, and atracurium 0.1-mg kg^−1^ every 20–30 min.

### 2.6. Block Technique

The work solutions were arranged in 2 syringes; each contained 20 ml of bupivacaine (0.25%). By the finish of the operation and earlier retrieval from general anesthesia, any blocks have been performed via a high-frequency US probe active array L12-4 (8–13 MHz) of a US device (*Philips ClearVue 350, Philips Healthcare, Andover MA01810, USA*) and a 22-gauge, 50-mm echogenic needles (Stimuplex D; B Braun, Germany).

For the US-directed QLB group, the case was located in the side location, and skin sterilizations have been performed via povidone-iodine. A high-frequency linear probe was then located above the iliac crest to identify three layers' 3 abdomen barrier muscles. First, transverse abdominis was outlined posteriorly till the transverse aponeurosis appeared, then the probe sloped a little caudal to improve transverse aponeurosis' appearances. Next, QL was recognized medial to the aponeurosis of transverse abdominal muscles. The needle was then injected from superfrontal to posteroinferior and progressive via the inplane method till the needle tip touched the anterolateral edge of the QL at its junction with the transversalis fascia. After negative aspirations (to reject intravascular injections), the precise needle location was approved by hydrodissection via 1 mL of saline. Then, 20 mL of 0.25% bupivacaine was utilized. The same method was achieved on the other side.

After executing the blocks, anesthesia was stopped, and tracheal extubating was performed after the case satisfied the extubating criteria. Then, cases were transmitted to the postanesthetic care unit (PACU), where they were discharged from the PACU; after that, an adapted Aldrete score was ≥ 9. VAS was utilized to measure postoperative pains. According to the protocol, all cases given analgesics succeeded (paracetamol 1 gm IV infusion/8 hrs and ketorolac 30-mg IM/12 hrs). Furthermore, postoperative rescue analgesia with morphine sulfate IV was received by patients with VAS >4 at a bolus dosage of 3-mg increments with a greatest amount of 15 mg/4 h or 45 mg a day.

### 2.7. Parameters and Outcomes

The primary outcome was the collective morphine consumption on the 1st day postoperatively. Secondary end points involve postoperative pains, evaluated via VAS score at 30 minutes, 2, 4, 6, 12, and 24 hours postoperatively, interval to the 1^st^ analgesic demand described as the period from recovery and the 1^st^ morphine dose.

## 3. Statistical Analysis

The sample size was estimated via the G power package 3.1.9.2, with total opioid consumption between the two groups as the primary outcome. They were preceding analogous research [9] that established that the effect size amongst both groups was supposed to be considerable of 1.12 and determined that 42 cases (21 case/group) would deliver a power of 95% with a type-I error rate of 0.05. But, we allocated 50 cases (25 cases/group) to balance data loss. The gathered data were statistically analyzed via the SPSS-22 package (IBM Inc, USA). Data have been examined for normality via the Shapiro–Wilks testing. Numerical variables like age, height, body mass, and BMI have a normal distribution and have been presented as mean ± standard deviation (SD). A nondependent *t*-testing was utilized to match the mean values of the two groups. Other variables have nonnormal distribution and are introduced as the median and interquartile range (IQR); the Mann–Whitney U testing has been utilized to test for significance. Qualitative data have been introduced as numbers and percentages, and the chi-squared testing has been utilized to test for significance. A 2-sided *P* value of <0.05 was guided to have statistical significance.

## 4. Results

**Figure 1:**
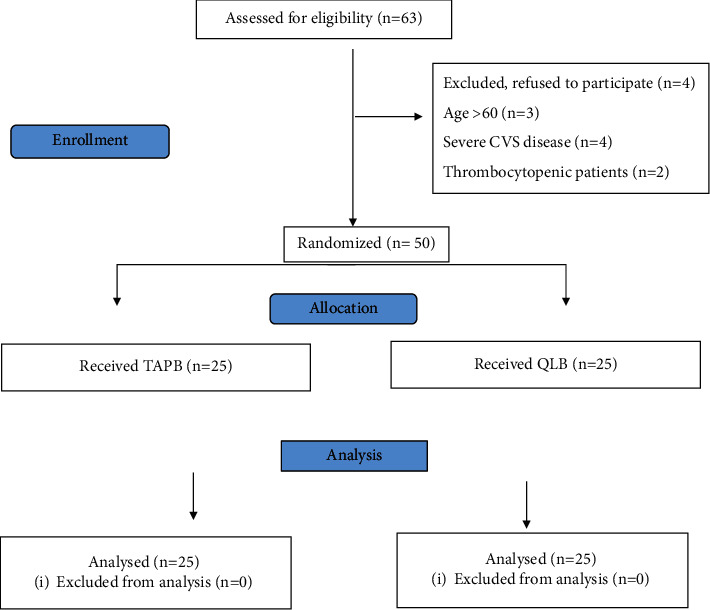
Consort flow diagram of the study population. *n*, number; TAPB, transversus abdominis plane block; QLB, quadratus lumborum block.

Sixty-three patients were admitted to the general surgery and gynecological departments in Fayoum University Hospital between July 2019 and February 2020 prepared for elective laparoscopic abdomen operations. Four cases were not accepted to contribute, and nine cases were excepted by exclusion criteria (age >60 years (*n* = 3), severe CVS disorder (*n* = 4), and thrombocytopenic patients (*n* = 2)). Fifty patients were finally analyzed in the two groups: the TAPB group and QLB 1 group (Figure 1).

**Figure 2:**
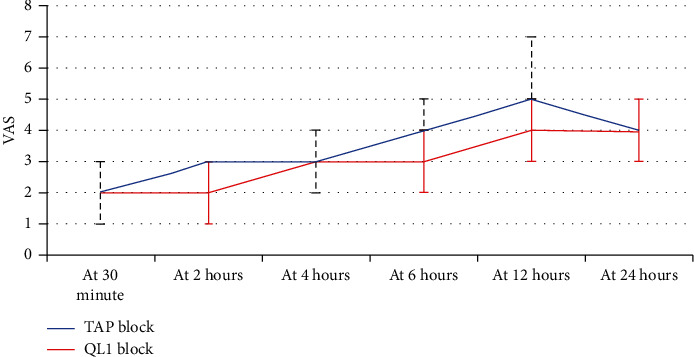
VAS scores among different study groups.

A nonsignificant change was found in age, spinal level, body mass, or parity (Table 1). There was a significant change in the median of cumulative morphine consumptions in the 1st day postoperatively between the QLB group and the TAPB group (3 mg [3, 6] vs. 6 mg [6, 9]; *p* < 0.0001) as shown in Table 2. The time passed before the 1^st^ added analgesic necessity was significantly high in the QLB group than in comparison to the TAPB group (17 h [ 12,24] vs. 8 h [6,24]; *p*=0.001), as shown in Table 2. The VAS pain scoring was significantly low in the QLB group compared to the TAPB group, as shown in Table 3 and Figure 2.

## 5. Discussion

In our study, we found that a QL block offers better postoperative analgesia. In addition, the QL block had a longer time required for the first analgesic requirement, lesser morphine dose required, and lower VAS score in these patients.

Current literature on the QLB reveals four different methods [10]. The approach followed during this study was the QL1 (lateral) block as this technique is comparatively safe and effective.

Ueshima et al. revealed that although there are four different approaches for this technique, the best approach is still to be tested depending on the surgery. [10].

Fifty cases have been involved in the ultimate analysis. They were arbitrarily assigned into two similar groups with 25 patients each, and the collective morphine consumptions in the 1^st^ day postoperatively were measured. The VAS score was also measured immediately postoperatively, at 30 min, 2, 4, 6, 12, and 24 hours. The time of the first analgesic request was reported as well.

The topographically broader field of action (T6 to L1) and a more extended period of pain relief make it more significant to TAPB in providing postoperative pain relief. [7, 11].

Our results are in line with the findings reported by Baidya et al. [12], who achieved transmuscular QL block on pediatric patients who underwent pyeloplasty. They said that it was correlated with good postoperative analgesia. Murouchi [13] utilized a bilateral QL intramuscular block on pediatric patients who underwent laparoscopic appendicectomy and recorded that it was related to adequate postoperative analgesia.

Yousef matched TAP and QL blocks on females who experienced entire abdominal hysterectomy [9]. Opioid necessity was lesser in the QLB group. VAS scoring was also significantly higher in the TAPB group. Thus, the results of the study were in line with our results in all means.

Öksüz et al. compared both blocks on pediatric patients who underwent orchidopexy or unilateral inguinal hernia repair. The opioid necessity within the first 24 h postoperatively was significantly lesser in the QLB group. FLACC scores were also lower in the QLB group. [14].

Our results are also in agreement with Blanco et al. [11] who concluded that QLB was better than TAPB after cesarean sections as it was accompanied by a more extended analgesic period (above one day), lesser opioid consumptions, and broader spread of analgesia. TAPB influenced T10 to T12 dermatomes while QLB covered from T7 to T12 dermatomes. They clarified their findings by the distance of local anesthetic medications moreover into the paravertebral cavity or in the thoracolumbar plane (which involves mechanoreceptors and a higher-density network of sympathetic fibers); this is widespread with the QLB-formed analgesia for somatic and visceral pains [11].

The coverage of local anesthetics throughout QLB to the paravertebral cavity was informed by Carney et al. [15] who concluded that single-shot QLB covered the dermatome segments from T4 to L2.

Murouchi et al. [7] studied the association between the local anesthetic blood levels and the type-2 QLB and TAPB effectiveness in adults. They revealed that the local anesthetic blood levels were high in TAPB compared to the type-2 QLB, but the analgesic influence was better with the type-2 QLB than with TAPB. This consequence was elucidated via the subsequent, throughout QLB, some of the managed medications are believed to move from the intermuscular cavity into the paravertebral cavity, which is full of adipose tissues, and the local tissues perfusions of the fatty tissues is low, which causes lower absorption rapidity of a local anesthetic to blood. [16].

Both techniques showed nonsignificant complications either due to the method utilized as the injection site usually is easily identifiable using ultrasound guidance with no proximity of major blood vessels or nerves or due to the postoperative morphine utilized as both techniques have an opioid-sparing effect. Regarding sedation scores, both methods showed no significant difference.

Our limitation was the lack of comparability because of the limited number of studies in the literature comparing both techniques after laparoscopy.

We recommend a more extended follow-up with larger sample sizes in the upcoming study to measure chronic pains management's effect and use a higher concentration of bupivacaine (0.375 and 0.5%) for a more intense block and a more extended period of postoperative analgesia.

## 6. Conclusion

In comparison to the TAPB, the QL block delivers more successful pain relief, has an extended period of analgesic actions, extends interval to 1^st^ analgesic necessity, is accompanied with lesser morphine consumptions, and may be utilized in multimodal analgesia and opioid-sparing regimens after that laparoscopic operation.

## Figures and Tables

**Table 1:** The patient's characteristics and operative data.

	TAB block (*N* = 25)		QL block (*N* = 25)		*P* value
	Mean	SD	Mean	SD	
Age (years)	33.2	9.1	32.7	8.4	0.860
Weight (kg)	68.9	7.4	66.3	8.5	0.252
Height (m)	1.6	0.1	1.6	0.1	0.356
BMI	26	2.8	25.5	2.9	0.531
	*N*	%	*N*	%	*P* value
Gender					
Male	4	16.0%	4	16.0%	1.000
Female	21	84.0%	21	84.0%	
ASA					
1(Normal healthy patient)	21	84.0%	24	96.0%	0.349
2(Mild systemic disorder)	4	16.0%	1	4.0%	
Operation					
Lap inguinal hernia	2	8.0%	3	12.0%	0.815
Lap appendectomy	3	12.0%	5	20.0%	
Lap missed IUCD extraction	13	52.0%	11	44.0%	
Lap ovarian vein ligation	7	28.0%	6	24.0%	

*Note.* Variables are reported as mean ± SD or number and percent. QL, quadratus lumborum; TAP, transversus abdominis plane; BMI, body mass index; N, number; IQR, interquartile range.

**Table 2:** Comparison of analgesic requirements on the 1st day among different study groups.

	TAB block (*N* = 25)		QL block (*N* = 25)		*P* value
	N	%	N	%	
Analgesics request					
Yes	18	72.0%	14	56.0%	0.239
No	7	28.0%	11	44.0%	
	Median	IQR	Median	IQR	*P* value
Time of first analgesic dose(hours)	8	(6–24)	17	(12–24)	**0.001** ^*∗*^
Cumulative morphine dose (mg)	6	(6–9)	3	(3–6)	**<0.0001** ^*∗*^

QL, quadratus lumborum; TAP, transversus abdominis plane; N, number; IQR, interquartile range.

**Table 3:** VAS scores among different study groups.

	TAB block (*N* = 25)		QL1 block (*N* = 25)		*P* value
	Median	IQR	Median	IQR	
VAS at 30 min. postoperative	2	(2-3)	2	(2-2)	0.084
VAS at 2 hours	3	(3-3)	2	(2-3)	**0.004** ^*∗*^
VAS at 4 hours	3	(3-4)	3	(3-3)	**0.008** ^*∗*^
VAS at 6 hours	4	(4-5)	3	(3-4)	**<0.0001** ^*∗*^
VAS at 12 hours	5	(4–6)	4	(3-4)	**<0.0001** ^*∗*^
VAS at 24 hours	4	(4-5)	4	(3-4)	**0.026** ^*∗*^

QL, quadratus lumborum; TAP, transversus abdominis plane; N, number; IQR, interquartile range.

## Data Availability

The datasets used and analyzed during the current study are available from the corresponding author on reasonable request.

## Abbreviations

QLB: Quadratus lumborum block
TAPB: Transversus abdominis plane block.
